# *Hedyotis diffusa* Willd Inhibits Colorectal Cancer Growth *in Vivo* via Inhibition of STAT3 Signaling Pathway

**DOI:** 10.3390/ijms13056117

**Published:** 2012-05-18

**Authors:** Qiaoyan Cai, Jiumao Lin, Lihui Wei, Ling Zhang, Lili Wang, Youzhi Zhan, Jianwei Zeng, Wei Xu, Aling Shen, Zhenfeng Hong, Jun Peng

**Affiliations:** 1 Academy of Integrative Medicine Biomedical Research Center, Fujian University of Traditional Chinese Medicine, 1 Huatuo Road, Minhou Shangjie, Fuzhou 350108, China; E-Mails: caiqiaoyan@fjtcm.edu.cn (Q.C.); linjiumao@gmail.com (J.L.); 282117657@qq.com (L.W.); 391118966@qq.com (L.Z.); 987131517@qq.com (L.W.); 691306289@qq.com (Y.Z.); zjwtcm@qq.com (J.Z.); 277708930@qq.com (A.S.); zfhong1953@163.com (Z.H.); 2 Fujian Key Laboratory of Integrative Medicine on Geriatrics, Fujian University of Traditional Chinese Medicine, 1 Huatuo Road, Minhou Shangjie, Fuzhou 350108, China; 3 Department of Pharmacology, Fujian University of Traditional Chinese Medicine, 1 Huatuo Road, Minhou Shangjie, Fuzhou 350108, China; E-Mail: xwfjtcm@sina.com

**Keywords:** *Hedyotis diffusa* Willd, Chinese medicine, colorectal cancer, STAT3 pathway, apoptosis, proliferation

## Abstract

Signal Transducer and Activator of Transcription 3 (STAT3), a common oncogenic mediator, is constitutively activated in many types of human cancers; therefore it is a major focus in the development of novel anti-cancer agents. *Hedyotis diffusa* Willd has been used as a major component in several Chinese medicine formulas for the clinical treatment of colorectal cancer (CRC). However, the precise mechanism of its anti-tumor activity remains largely unclear. Using a CRC mouse xenograft model, in the present study we evaluated the effect of the ethanol extract of *Hedyotis diffusa* Willd (EEHDW) on tumor growth *in vivo* and investigated the underlying molecular mechanisms. We found that EEHDW reduced tumor volume and tumor weight, but had no effect on body weight gain in CRC mice, demonstrating that EEHDW can inhibit CRC growth *in vivo* without apparent adverse effect. In addition, EEHDW treatment suppressed STAT3 phosphorylation in tumor tissues, which in turn resulted in the promotion of cancer cell apoptosis and inhibition of proliferation. Moreover, EEHDW treatment altered the expression pattern of several important target genes of the STAT3 signaling pathway, *i.e.*, decreased expression of Cyclin D1, CDK4 and Bcl-2 as well as up-regulated p21 and Bax. These results suggest that suppression of the STAT3 pathway might be one of the mechanisms by which EEHDW treats colorectal cancer.

## 1. Introduction

Colorectal cancer (CRC) is a major health problem in Western societies representing the second most common cause of cancer-related death. Both the yearly incidence and the mortality rate also increase steeply with age [1]. Although approximately 70–80% of patients are eligible for curative surgical resection at the time of diagnosis, about 50% of all newly diagnosed patients ultimately develop metastatic disease [2]. In the last decade, irinotecan, oxaliplatin and the standard fluorouracil-based chemotherapy regimens have set the new benchmark of survival for patients with metastatic CRC [3]. However, such treatments are also often coupled with serious toxicity and side effects such as anemia, leucopenia, thrombocytopenia and peripheral neuropathy [4]. Therefore, it is essential to find a safer and more effective way to treat CRC. The traditional Chinese medicine (TCM) has held and played an important role in primary health care in China and has been recently recognized by Western countries as an abundant source of novel lead molecules for modern drug discovery. Clinical practice has also shown that many traditional Chinese herbal medicines have antitumor activity, which sheds light on new therapeutic strategies for cancer treatment [4,5].

Signal transducer and activator of transcription 3 (STAT3) plays an important role in cell proliferation and apoptosis [6]. The activation of STAT3 is mediated by phosphorylation of tyrosine residue 705 in the Src homology-2 domain (SH2) induced by a wide range of cytokine receptor-associated kinases, growth factor receptor tyrosine kinases, and non-receptor tyrosine kinases [7–9]. In the cytoplasm, phosphorylated STAT3 proteins dimerize and translocate to the nucleus where they regulate the expression of various critical genes involved in cell proliferation and survival [10,11]. Constitutive activation of STAT3, resulting in an unregulated increase in cell proliferation and reduction in cell apoptosis, is strongly correlated with the development of numerous types of cancer including CRC [12–15]. Therefore, inhibiting cell proliferation and/or promoting apoptosis by suppression of STAT3 activation has been a major focus in the development of anti-cancer drugs.

*Hedyotis diffusa* Willd (HDW), belonging to the Rubiaceae family, is a medicinal herb widely distributed in northeast Asia. As a well-known traditional Chinese herbal medicine, HDW has long been widely applied in the treatment of inflammation-related diseases, such as appendicitis, urethritis and bronchitis [16,17]. Modern pharmacological studies proposed that HDW displays therapeutic effects in the treatment of liver, lung, colon, brain and pancreatic cancers [18–20]. Our previous studies demonstrated that HDW can promote the apoptosis of human colon carcinoma cells and inhibit tumor angiogenesis *in vitro* [21,22]. However, the precise mechanism of its anti-cancer activity *in vivo* remains largely unknown. In order to further elucidate the anti-cancer mechanism of HDW, in the present study we evaluated the efficacy of HDW against tumor growth *in vivo* in the CRC mouse xenograft model and investigated the underlying molecular mechanisms.

## 2. Materials and Methods

### 2.1. Materials and Reagents

Dulbecco’s modified Eagle’s medium (DMEM), fetal bovine serum (FBS), penicillin-streptomycin, trypsin-EDTA, Trizol reagent were purchased from Invitrogen (Carlsbad, NM, USA). SuperScript II reverse transcriptase was provided by Promega (Madison, WI, USA). PCNA, Bcl-2, Bax, P21, cyclinD1, CDK4 antibodies, horseradish peroxidase (HRP)-conjugated secondary anti-bodies were obtained from Cell Signaling (Beverly, MA, USA). pcna assay, TUNEL assay kit was purchased from R&D Systems (Minneapolis, MN, USA), VECTASTAIN Elite ABC kit was provided by VECTOR Laboratories (Burlingame, CA, USA). All the other chemicals used, unless otherwise stated, were obtained from Sigma Chemicals (St. Louis, MO, USA).

### 2.2. Preparation of Ethanol Extract from *Hedyotis diffusa* Willd

Authentic plant material was purchased from Guo Yi Tang Chinese Herbal medicine store, Fujian, China. The original herb was identified as *Hedyotis diffusa* Willd (HDW) by Dr. Wei Xu at Department of Pharmacology, Fujian University of Traditional Chinese Medicine, China. Ethanol extract of HDW (named EEHDW) was prepared as previously described [21]. Briefly, 500 g of HDW was extracted with 5000 mL of 85% ethanol using a refluxing method and filtered. The ethanol solvent was then evaporated on a rotary evaporator (Yarong, Model RE-2000, Shanghai, China). The resultant solution was concentrated to a relative density of 1.05, and the dried powder of EEHDW was obtained by a spraying desiccation method using a spray dryer (Buchi, Model B-290, Flawil, Switzerland). The working concentrations of EEHDW were made by dissolving the extract in saline to a concentration of 0.6 g/mL.

### 2.3. HPLC Analysis

**Figure 1:**
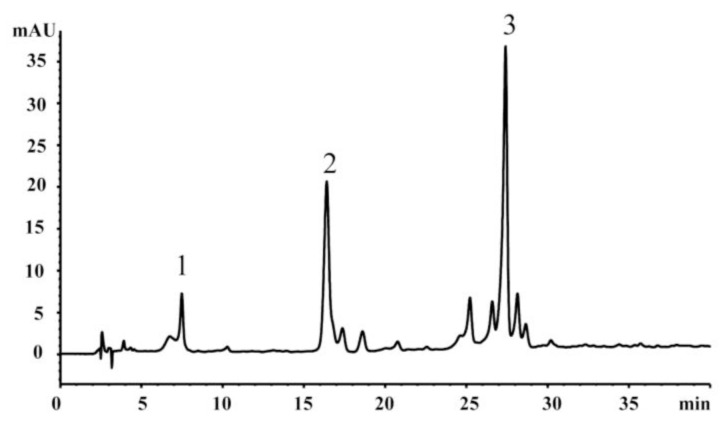
Chromatographic profile of ethanol extract of *Hedyotis diffusa* Willd (EEHDW). In order to control the quality of EEHDW, the peak areas of 1, 2, 3 should be in the optimum ratio of 1:6.4:9.2. An Agilent 1200 series HPLC system with DAD detector and a Sepax Sapphire-C_18_ column was used. The mobile phase consisted of solvent A (methanol) and solvent B (water). The gradient procedure was used as follows: 20–30% A at 0–15 min and 40–60% A at 15–40 min. The flow rate was 1.0 mL/min and the column temperature was set at 30 °C.

Chromatographic fingerprint was employed as quality control for different batches [23]. Samples were analyzed on an Agilent 1200 HPLC system (Agilent, Santa Clara, CA, USA) using a Sepax Sapphire-C_18_ column (250 mm × 4.60 mm, 5 μm). The absorbance was measured at 355 nm. The mobile phase consisted of solvent A (methanol) and solvent B (water) at a flow rate of 1 mL/min with an injection volume of 10 μL. The gradient procedure was used as follows: 20–30% A at 0–15 min and 40–60% A at 15–40 min. Based on the fingerprint as shown in Figure 1, we established an optimum and easily controlled procedure for preparing the EEHDW extract as mentioned above. If the peak areas of 1, 2, and 3 are in an optimum ratio of 1:6.4:9.3, all results from the study would readily be reproducible.

### 2.4. Cell Culture

Human colon carcinoma HT-29 cells were obtained from American Type Culture Collection (ATCC, Manassas, VA, USA). Cells were grown in DMEM containing 10% (v/v) FBS, 100 units/mL penicillin and 100 μg/mL streptomycin in a 37 °C humidified incubator with 5% CO_2_.

### 2.5. Animals

Athymic BALB/c nu/nu male mice (with an initial body weight of 20–22 g) were obtained from Shanghai SLAC Laboratory Animal Co., Ltd. (Shanghai, China) and housed under pathogen-free conditions with a 12 h light/dark cycle. Food and water were given *ad libitum* throughout the experiment. All animal treatments were strictly in accordance with international ethical guidelines and the National Institutes of Health Guide concerning the Care and Use of Laboratory Animals, and the experiments were approved by the Institutional Animal Care and Use Committee of Fujian University of Traditional Chinese Medicine.

### 2.6. *In Vivo* Nude Mouse Xenograft Study

HT-29 cells were grown in culture and then detached by trypsinization, washed, and resuspended in serum-free DMEM. 1.5 × 10^6^ of cells mixed with Matrigel (1:1) were subcutaneously injected in the right flank area of athymic nude mice to initiate tumor growth. After 3 days of xenograft implantation, mice were randomized into two groups (*n* = 12) and given intra-gastric administration of 6 g/kg of EEHDW or saline daily, 5 days a week for 16 days. Body weight and tumor growth were measured every two days. Tumor growth was determined by measuring the major (L) and minor (W) diameter with a caliper. The tumor volume was calculated according to the following formula: tumor volume = π/6 × L × W^2^. At the end of experiment, the animals were anaesthetized with pelltobarbitalum natricum, and the tumor tissue was removed and weighed.

### 2.7. Immunohistochemical Staining for PCNA

Tumor samples were fixed in 10% buffered formalin for 12 h, and subsequently processed conventionally for paraffin-embedded tumor sections (4 μm thick). The tumor sections were then deparaffinized and rehydrated via treatment with a series of xylenes and graded alcohols. Endogenous peroxidase activity of the sections was quenched by incubating in water containing 0.3% H_2_O_2_ for 30 min. Immunohistochemical staining was performed using the VECTASTAIN elite ABC kit according to the manufacturer’s instructions. Briefly, after blocking non-specific proteins with normal serum in PBS (0.1% Tween 20), the sections were treated with PCNA antibody (at a dilution of 1:200) for 1 h. The sections were incubated with a biotinylated anti-rabbit IgG antibody for 30 min and then treated with the ABC reagent for 30 min. They were finally treated with DAB for 10 min. After the sections were washed and air-dried, cover slips were applied to the sections using permount slide mounting medium. PCNA was quantified by counting respective positive cells and total number of cells in five high-power fields (×400) randomly selected in each slide, and the average proportion of positive cells in each field were counted using the true color multi-functional cell image analysis management system (Image-Pro Plus, Media Cybernetics, Bethesda, MD, USA).

### 2.8. *In Situ* Apoptosis Detection by TUNEL Staining

The 4-μm-thick sections of tumor samples were analyzed by TUNEL staining using TumorTACS *in situ* Apoptosis kit (R&D Systems, Minneapolis, MN, usa). Apoptotic cells were counted as DAB-positive cells (brown stained) at five arbitrarily selected microscopic fields at a magnification of 400×. TUNEL-positive cells were counted as a percentage of the total cells.

### 2.9. RNA Extraction and RT-PCR Analysis

Total RNA was isolated from fresh tumor with Trizol reagent. Oligo(dT)-primed RNA (1 μg) was reverse transcribed with SuperScript II reverse transcriptase according to the manufacturer’s instructions. The obtained cDNA was used to determine the mRNA amount of Bcl-2, Bax, p21, Cyclin D1 and CDK4 by PCR with Taq DNA polymerase (Fermentas). GAPDH was used as an internal control. The primers used for amplification of Bcl-2, Bax, p21, Cyclin D1, CDK4 and GAPDH transcripts are as follows: Bcl-2 forward 5′-CAG CTG CAC CTG ACG CCC TT-3 and reverse 5′-GCC TCC GTT ATC CTG GAT CC-3′; Bax forward 5′-TGC TTC AGG GTT TCA TCC AGG-3′ and reverse 5′-TGG CAA AGT AGA AAA GGG CGA-3′; p21 forward 5′-GCG ACT GTG ATG CGC TAA TGG-3′ and reverse 5′-TAG AAA TCT GTC ATG CTG GTC TGC-3′; Cyclin D1 forward 5′-TGG ATG CTG GAG GTC TGC GAG GAA-3′ and reverse 5′-GGC TTC GAT CTG CTC CTG GCA GGC-3′; CDK4 forward 5′-CAT GTA GAC CAG GAC CTA AGC-3′ and reverse 5′-AAC TGG CGC ATC AGA TCC TAG-3′; GAPDH forward 5′-GT CAT CCA TGA CAA CTT TGG-3′ and reverse 5′-GA GCT TGA CAA AGT GGT CGT-3′.

### 2.10. Preparation of Tumor Homogenate and Western Immunoblotting

Three tumors were selected randomly from control or EEHDW group, homogenized in nondenaturing lysis buffer using homogeniser and centrifuged at 15,000× g for 15 min followed by determination of protein concentration in supernatants. Equal protein per lysate was resolved on Tris-glycine gel, transferred onto PVDF membrane, and blocked for 2 h with 5% nonfat dry milk. Membranes were incubated with desired primary antibody Bcl-2, Bax, p21, Cyclin D1, CDK4, and β-actin (at a dilution of 1:1000) overnight at 4 °C and then with appropriate HRP-conjugated secondary antibody followed by enhanced chemiluminescence detection.

### 2.11. Statistical Analysis

Data were presented as mean ± SD for the indicated number of independently performed experiments. The statistical analysis between groups was carried out using the Student’s *t* test and *p* < 0.05 was considered to be statistically significant.

## 3. Results

### 3.1. EEHDW Inhibits Tumor Growth in CRC Xenograft Mice

**Figure 2:**
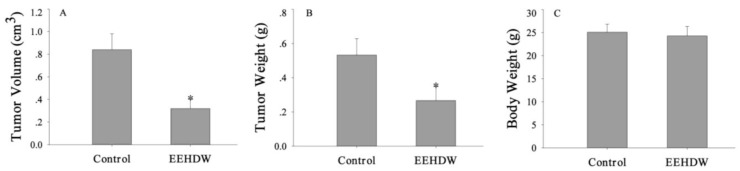
Effect of EEHDW on tumor growth in CRC xenograft mice. After tumor development, the mice were given intra-gastric administration of a 6 g/kg/day dose of EEHDW or saline daily, 5 days a week for 16 days. Tumor volume (**A**), tumor weight (**B**) and body weight (**C**) were measured at the end of the experiment. Data shown are averages with SD (error bars) from 12 individual mice in each group. ^*^ *p* < 0.01, *versus* controls.

The anti-tumor effect of EEHDW *in vivo* was evaluated by measuring tumor volume and weight in CRC xenograft mice, while its adverse effect was determined by measuring the body weight gain. As shown in Figures 2A,B, administration of EEHDW decreased tumor volume and weight by 52% and 45%, respectively, compared with control. However, EEHDW treatment had no effect on the body weight gain in experimental animals (Figure 2C). These data suggest that EEHDW can inhibit colorectal tumor growth *in vivo*, without apparent signs of toxicity.

### 3.2. EEHDW Inhibits Cell Proliferation and Induces Apoptosis in CRC Xenograft Mice

**Figure 3:**
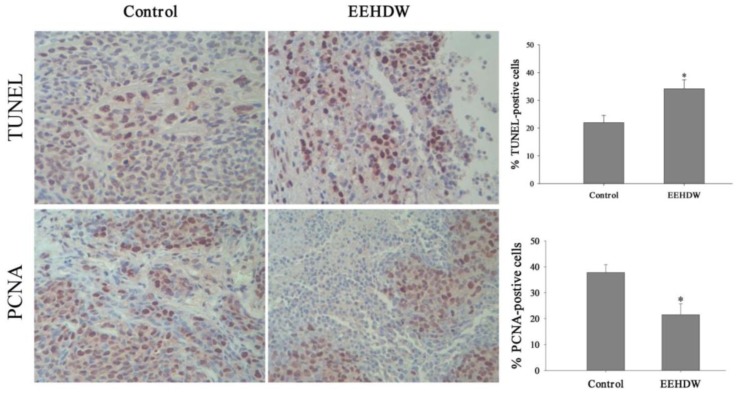
Effect of EEHDW on cell apoptosis and proliferation in CRC mice. At the end of the experiment, tumor tissues were processed for immunohistochemical (IHC) staining for TUNEL (top) or PCNA (bottom). Left: The photographs are representative images taken at a magnification of 400×. Right: Quantification of IHC assay is represented as percentage of positively-stained cells. Data shown are averages with SD (error bars) from 10 individual mice in each group. ^*^ *p* < 0.01, *versus* controls.

The *in vivo* effect of EEHDW on cell apoptosis and proliferation was determined by immunohistochemical (IHC) staining for TUNEL and PCNA. Data in Figure 3 showed 22.0 ± 2.59% and 34.2 ± 3.19% TUNEL-positive cells in control and EEHDW-treated mouse groups, and the percentage of PCNA-positive cells in control and EEHDW-treated mice was 37.8 ± 3.08% and 21.5 ± 4.22%, respectively. Taken together, it is demonstrated that EEHDW inhibits the proliferation of colorectal cancer cells and promotes cell apoptosis *in vivo*.

### 3.3. EEHDW Inhibits the Phosphorylation of STAT3 in CRC Xenograft Mice

**Figure 4:**
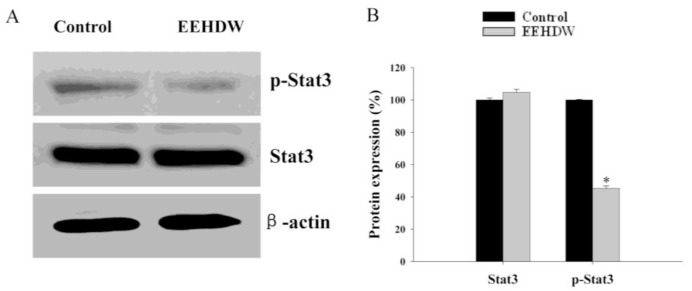
Effect of EEHDW on phosphorylation of STAT3 in CRC xenograft mice. (**A**) Three tumors were randomly selected from each group and the level of STAT3 phosphorylation in tumor tissues was determined by Western blot using an antibody that recognizes phosphorylated STAT3 at Tyr^705^. β-actin was used as the internal control. For each tumor sample, the Western blot assay was performed in triple. Data shown are representatives. (**B**) Densitometric analysis. The data were normalized to the mean protein expression of untreated control (100%). ^*^ *p* < 0.01, *versus* controls.

The activation of STAT3 is mediated by phosphorylation at tyrosine residue 705. We therefore examined the effect of EEHDW on STAT3 phosphoralytion in tumor tissue by Western blot analysis using antibody that recognizes STAT3 phosphorylation at Tyr^705^. As shown in Figure 4, EEHDW inhibited the phosphorylation of STAT3 in CRC mice, whereas the level of non-phosphorylated STAT3 remained unchanged after EEHDW treatment, suggesting that EEHDW significantly suppresses the activation of STAT3 *in vivo*.

### 3.4. EEHDW Regulates the Expression of Bcl-2, Bax, p21, Cyclin D1 and CDK4 in CRC Xenograft Mice

**Figure 5:**
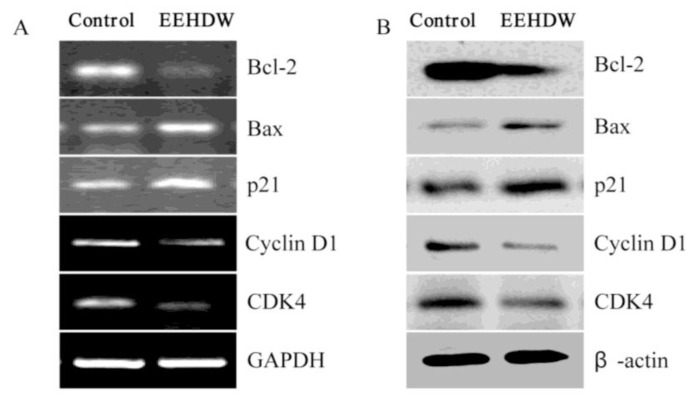

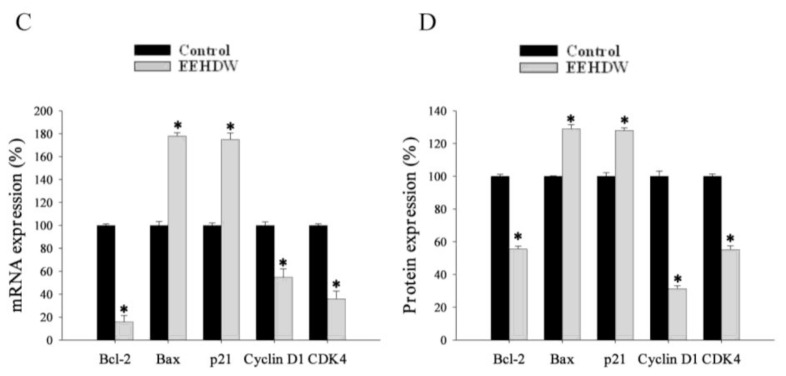
Effect of EEHDW on the expression of Bcl-2, Bax, p21, Cyclin D1 and CDK4 in CRC xenograft mice. (**A**,**B**) Three tumors were randomly selected from each group and the mRNA or protein expression levels of Bcl-2, Bax, p21, Cyclin D1, CDK4 were determined by RT-PCR (**A**) and Western blot analyses (**B**). GAPDH and β-actin were used as the internal controls for the RT-PCR or Western blotting, respectively. For each tumor sample, Western blot and RT-PCR analyses were was performed in triple. Data shown are representatives. (**C**,**D**) Densitometric analysis. The data were normalized to the mean mRNA (**C**) or protein (**D**) expression of untreated control (100%). ^*^ *p* < 0.01, *versus* controls.

To further explore the mechanism of EEHDW’s pro-apoptotic and anti-proliferative activities, we performed RT-PCR and Western Blot analyses to examine the mRNA and protein expression, respectively, of the pro-proliferative Cyclin D1, CDK4, anti-proliferative p21 and the pro-apoptotic Bax, anti-apoptotic Bcl-2. The results of the RT-PCR assay showed that EEHDW treatment profoundly reduced the mRNA expression of Bcl-2, p21, Cyclin D1, CDK4 but increased that of Bax and p21 in CRC mice (Figures 5A and C). Data from Western blot analysis showed that the pattern of protein expression was similar to the respective mRNA levels (Figures 5 B,D).

## 4. Discussion

To date, chemotherapy is one of the main therapeutic approaches for patients with advanced colorectal cancer (CRC), and 5-fluorouracil-based regimens are the standard treatment for these patients. However, drug resistance and cytotoxicity against normal cells limit the long-term use, so it is necessary to develop multi-target agents with minimal side effects [24]. Natural products, including Traditional Chinese Medicine (TCM), have relatively fewer side effects and have been used clinically for thousands of years to treat various types of diseases, including cancer [5,25]. As a well-known and important Chinese medicinal herb, *Hedyotis diffusa* Willd (HDW) has been used as a major component in several TCM formulas for the clinical treatment of various types of cancer, including CRC, without apparent adverse effect [26–28]. However, the precise mechanism of its anti-cancer activity remains largely unclear.

Cancer cells are characterized by an uncontrolled increase in cell proliferation and/or a reduction in cell apoptosis [29]. Bcl-2 family proteins are key regulators of apoptosis, functioning as either suppressors such as Bcl-2, or promoters such as Bax. The ratio of active anti- and pro-apoptotic Bcl-2 family members determines the fate of cells, and alteration of the ratio by aberrant expression of these proteins impairs the normal apoptotic program contributing to various apoptosis-related diseases including cancer [29,30]. Higher Bcl-2 to Bax ratios due to the overexpression of Bcl-2 or down-regulation of Bax expression are commonly found in cancers, which not only confers a survival advantage to the cancer cells but also causes resistance to chemo- and radio-therapies. Eukaryotic cell proliferation is primarily regulated by cell cycle. G1/S transition is one of the two main checkpoints of the cell cycle [31], which is responsible for initiation and completion of DNA replication. G1/S progression is strongly regulated by Cyclin D1 that exerts its function via forming an active complex with its CDK major catalytic partners (CDK4/6) [32]. An unchecked or hyperactivated Cyclin D1/CDK4 complex often leads to uncontrolled cell division and malignancy [33–36]. As a proliferation inhibitor, p21 protein plays a role in G1 arrest by binding to and inhibiting the activity of Cyclin-CDK complexes; and the decrease of p21 is associated with the promotion of tumor formation and a poor prognosis in many types of cancer [36,37]. The transcription factor STAT3 is essential for the regulation of cell apoptosis and proliferation. Phosphorylation of STAT3 at tyrosine 705 leads to its homodimerization, nuclear translocation and DNA binding, which in turn regulates the expression of various critical genes involved in cell proliferation and survival, such as the above-mentioned Bcl-2, Bax, p21, Cyclin D1 and CDK4 [10,11,38]. Constitutive activation of STAT3 facilitates an unregulated increase in cell proliferation and reduction in cell apoptosis resulting in the development of various cancers. Therefore, a re-balance of cell apoptosis and proliferation via regulating STAT3 pathway and its target gene expression has been a promising target for the development of anti-cancer therapies.

Using a CRC mouse xenograft model, in the present study we reported that the ethanol extract of HDW (EEHDW) could inhibit cancer growth *in vivo*, without any noticeable toxicity. In addition, EEHDW decreased the phosphorylation activation of STAT3 in CRC mice. Consequently, the inhibitory effect of EEHDW on STAT3 activation resulted in the suppression of cell proliferation and the induction of cell apoptosis. Moreover, EEHDW treatment profoundly reduced the expression of anti-apoptotic Bcl-2, pro-proliferative Cyclin D1 and CDK4, as well as increased the expression of anti-proliferative p21 and pro-apoptotic Bax *in vivo*.

## 5. Conclusions

In conclusion, for the first time we demonstrate that *Hedyotis diffusa* Willd inhibits colorectal cancer growth *in vivo* via promoting the apoptosis of cancer cells and inhibition of proliferation, which is mediated by the suppression of the STAT3 pathway. Our findings suggest that *Hedyotis diffusa* Willd may be a potential novel therapeutic agent for the treatment of cancers with constitutive activation of STAT3.

## Abbreviations

CRC: colorectal cancer
STAT3: signal transducer and activator of transcription 3
EEHDW: ethanol extract of *Hedyotis diffusa* Willd
DMSO: dimethyl sulfoxide
TCM: traditional Chinese medicine
TUNEL: terminal deoxynucleotidyl transferase-mediated dUTP nick end labeling
PCNA: proliferating cell nuclear antigen
